# Supplementary figures and images for: Drosophila host defense mechanisms against filamentous fungal pathogens with diverse lifestyles
Source: PLoS Pathog. 2026 Mar 23;22(3):e1013995. doi: 10.1371/journal.ppat.1013995 (PMC13035236; doi:10.1371/journal.ppat.1013995)

A

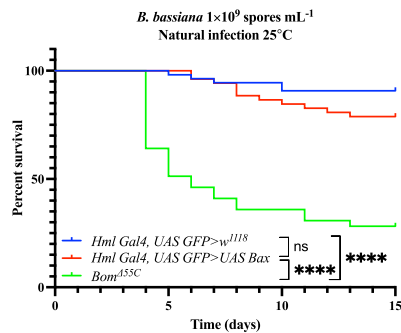

B

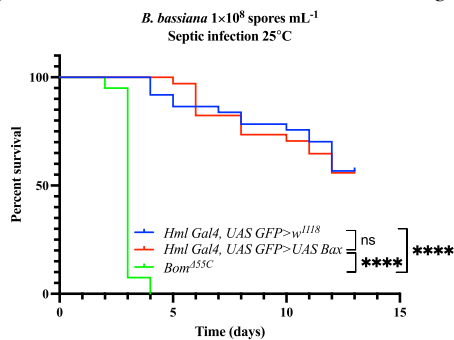

C

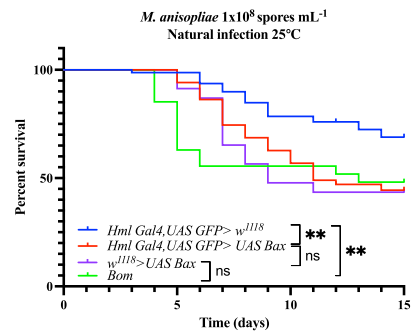

E

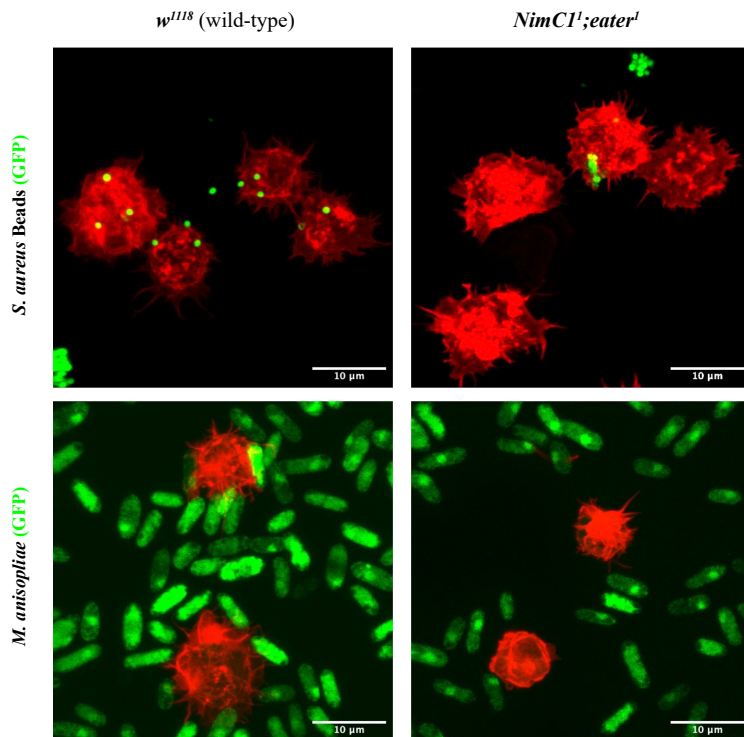

D

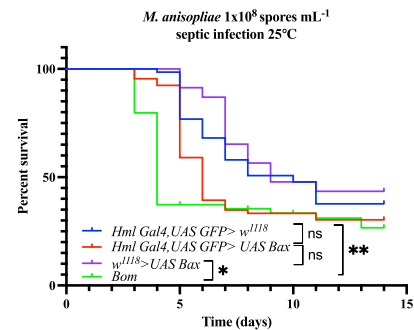

F

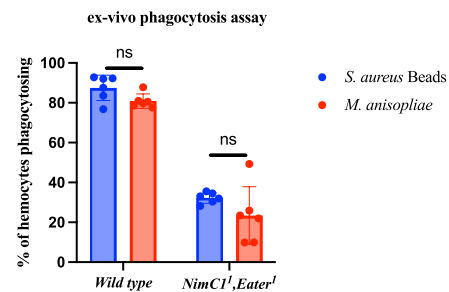

Supplement: S1 Fig — (A-D) Survival analysis of males lacking most plasmatocytes—due to Bax overexpression in plasmatocytes (HmlΔ-GAL4, UAS-GFP/ UAS-Bax) [103]—shows only a modest reduction in their ability to survive natural or septic injury infection with B. bassiana and M. anisopliae. Given the numerous roles of hemocytes in immunity and metabolism (Westlake et al., 2024b), these experiments cannot disentangle the specific contribution of hemocytes to antifungal defense. Both systemic and natural infections were carried out at 25 °C. BomΔ55C flies were included as a positive control. (E). Spores of M. anisopliae-GFP bind to wild-type but not NimC11;eater1 deficient plasmatocyte (stained in red with phalloidin). Plasmatocytes from third instar larvae were incubated in presence of M. anisopliae-GFP for 2 hours. Representative image are shown. (F) NimC11;eater1 deficient plasmatocytes display reduced ability to phagocyte or bind to spores of M. anisopliae. Plasmatocytes from third instar larvae were incubated in presence of M. anisopliae-GFP or bead of S. aureus for 2 hours. Note that this assay does not allow to distinguish if plasmatocytes bind to spores or uptake it internally. (PDF) [file ppat.1013995.s001.pdf]

**Figure S2**

**A**

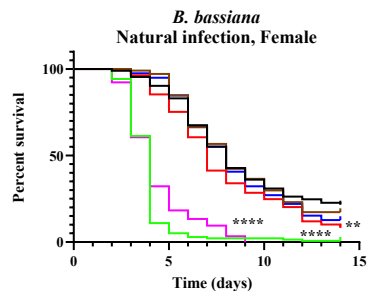

**B**

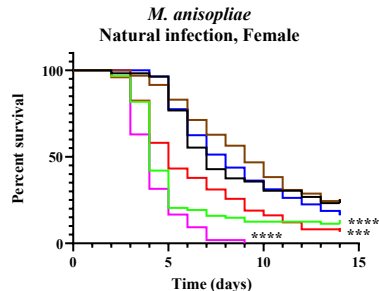

**C**

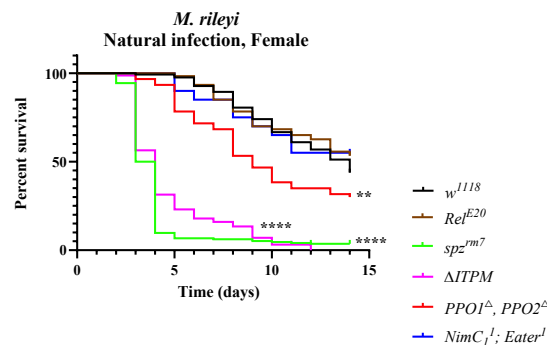

**D**

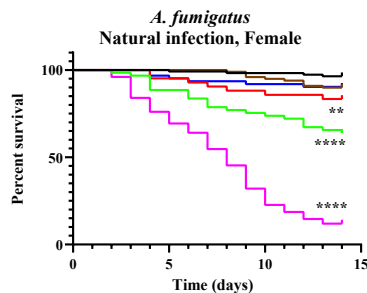

**E**

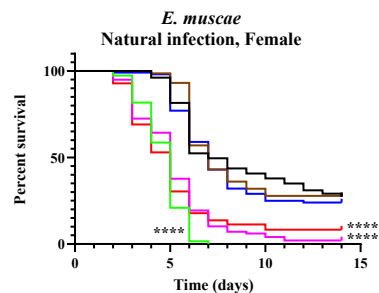

**F**

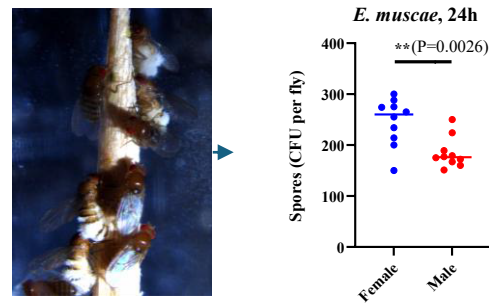

Supplement: S2 Fig — (A-E) Survival rate of the females shows that spzrm7 flies (P < 0.0001) and PPO1, PPO2 double mutant flies (P < 0.0001) are severely affected in their capacity to survive infection with the five fungi compared to wild-type w1118 flies. (F) The evaluation of spore concentration after the 24 h ejection from the fresh cadavers shows that more spores were produced from female cadavers than male cadavers (**, P < 0.01). For natural infection of E. muscae, a total of 10 female and 10 male fresh cadavers were used for one infection treatment by spore shower. Data were analyzed using the Log rank test and values are pooled data from at least three independent experiments. Full statistical details are available on S3 Table. Related to Fig 1. (PDF) [file ppat.1013995.s002.pdf]

**Figure S3**

**A**

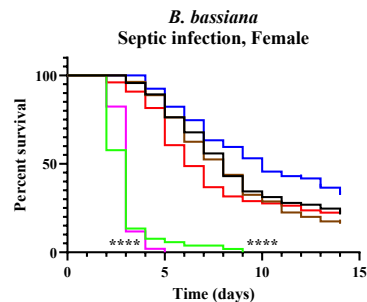

**B**

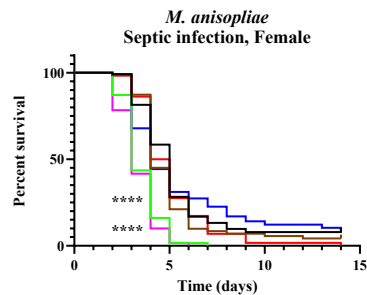

**C**

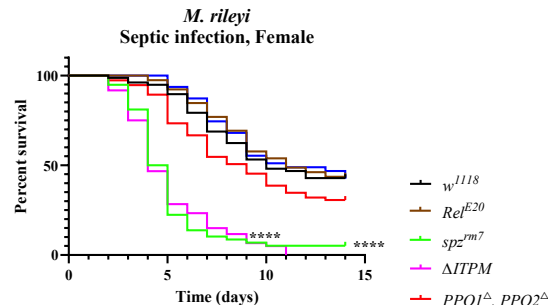

**D**

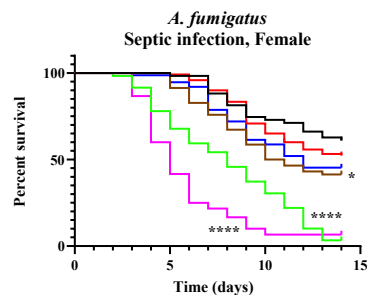

**E**

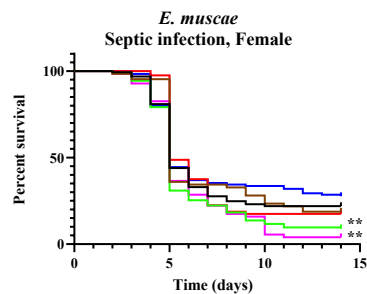

**F**

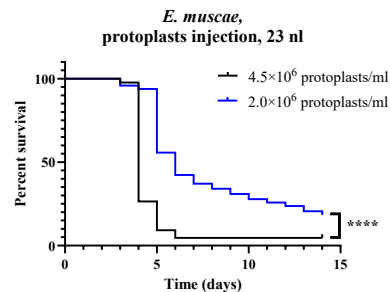

Supplement: S3 Fig — (A-C) Survival rate of females following pricking with spore suspensions show that spzrm7 flies (P < 0.0001) contribute most to surviving infection by the B. bassiana, M. anisopliae, and M. rileyi. (D) spzrm7 and to lower extent RelE20 females exhibited a strong susceptibility to infection with A. fumigatus (P < 0.0001). (E) No significant difference in survival of the four main modules upon infection with E. muscae by the injection of protoplasts compared to wild-type females. (F) Survival of w1118 flies upon infection with E. muscae showed that faster killing was found when injected with a higher concentration of protoplasts (****, P < 0.0001). Data were analyzed using the Log rank test and values are pooled data from at least three independent experiments. Full statistical details are available on S3 Table. Related to Fig 2. (PDF) [file ppat.1013995.s003.pdf]

**Figure S4**

**A**

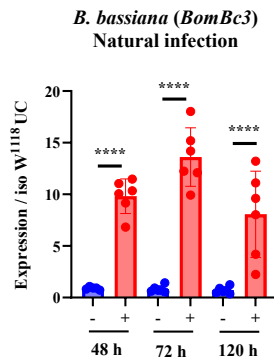

**B**

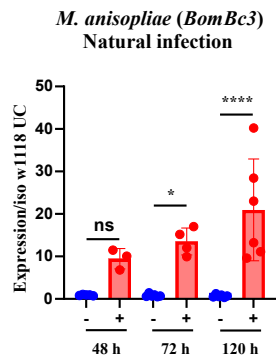

**C**

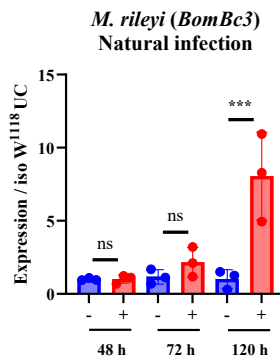

**D**

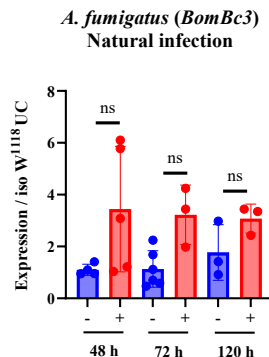

**E**

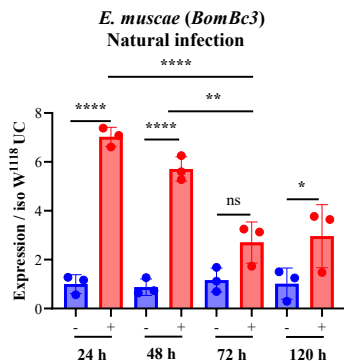

**F**

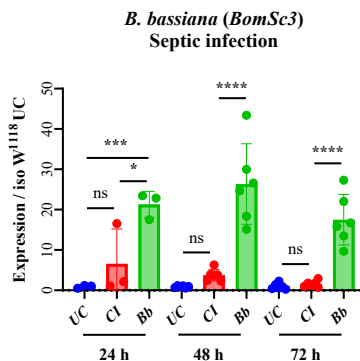

**G**

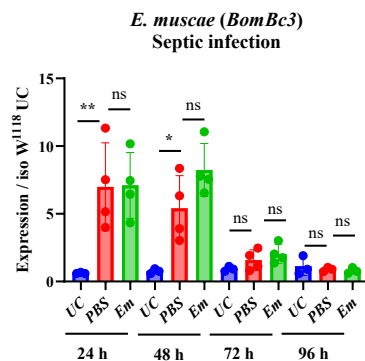

Supplement: S4 Fig — (A-C) Natural infection with M. anisopliae induced a highest expression of BomBc3, followed by B. bassiana and M. rileyi only induced the expression of BomBc3 at a late stage, at 120 h after infection. (D) Natural infection with A.fumigatus could not induce the expression of BomBc3. (E) Natural infection with E. muscae induced a low expression of BomBc3 at 24 h and 48 h after infection, then followed by a significant decrease. (F) Septic infection with B. bassiana spores activates higher BomBc3 expression compared to natural infection and clean injury with a needle. (G) Expression of BomBc3 after injection with E. muscae protoplasts was similar to the injection with PBS, indicating protoplasts cannot activate Toll signaling. Expression was normalized with w1118 UC set as a value of 1. Data were analyzed using One-Way ANOVA followed by Tukey’s multiple comparison tests. ns, P > 0.05; *, P < 0.05; **, P < 0.01; ***, P < 0.001; ****, P < 0.0001. Values represent the mean ± s.d. of at least three independent experiments. Full statistical details are available on S3 Table. Related to Fig 3. (PDF) [file ppat.1013995.s004.pdf]

**Figure S5**

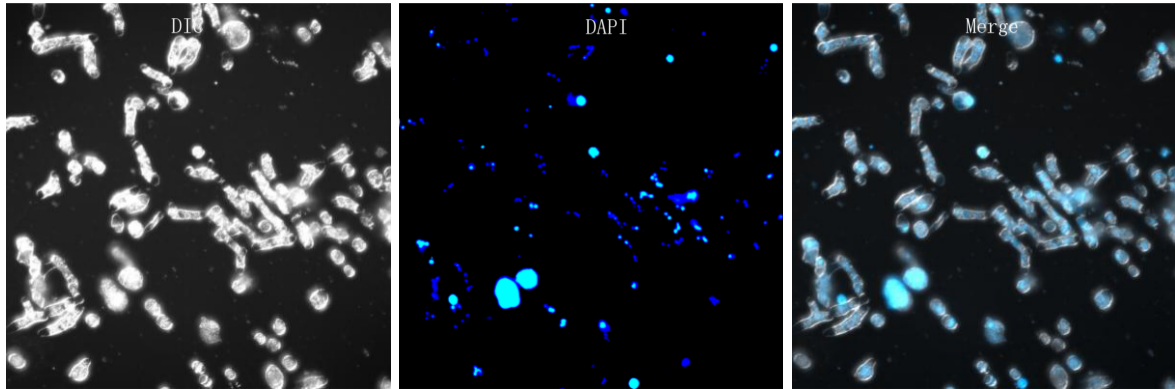

Supplement: S5 Fig — (PDF) [file ppat.1013995.s005.pdf]

# Figure S6

## A

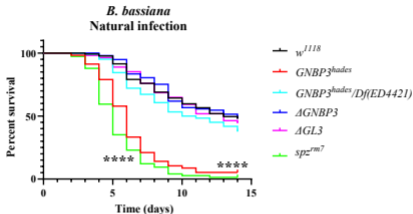

## B

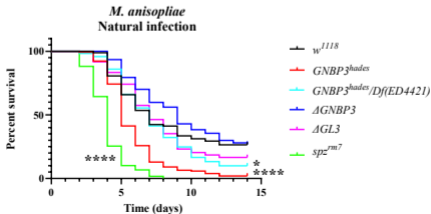

Supplement: S6 Fig — (A-B) Survival rate of male flies following infection with the two fungi shows that both GNBP3 and GNGP like 3 mutant flies are not susceptible or slightly susceptible to B. bassiana and M. anisopliae. Full statistical details are available on S3 Table. Related to Fig 5. (PDF) [file ppat.1013995.s006.pdf]

**Figure S7**

*w<sup>1118</sup>*

*spz<sup>rm7</sup>*

Day 3 post death

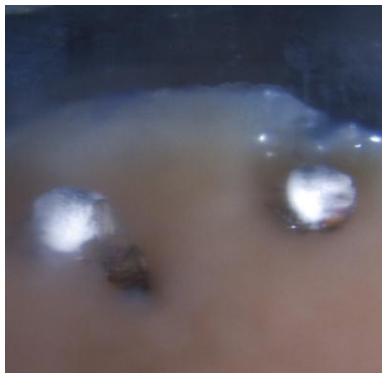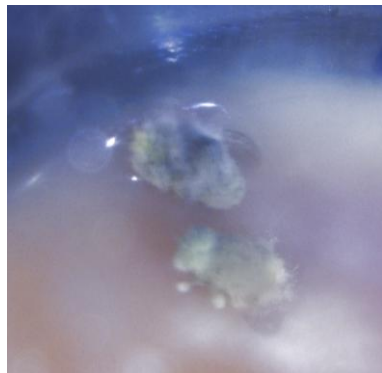

Day 6 post death

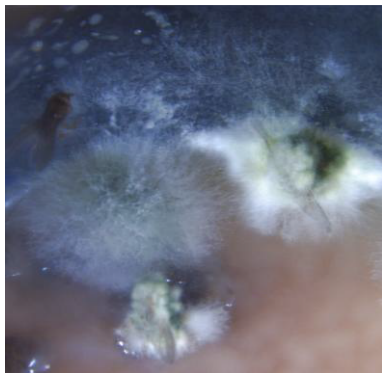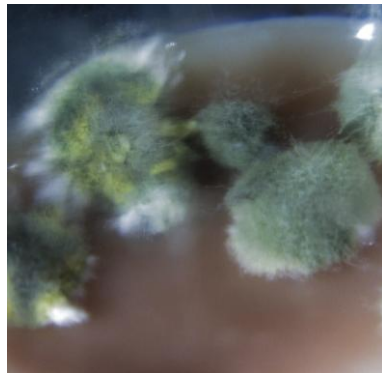

Supplement: S7 Fig — Metarhizium anisopliae extruding from cuticle and growing in spzrm7 cadavers are faster than that in wild-type cadavers. M. anisopliae can eventually disseminate through the whole flies both in wild-type and spzrm7 cadavers. Related to Fig 6. (PDF) [file ppat.1013995.s007.pdf]

**Figure S8**

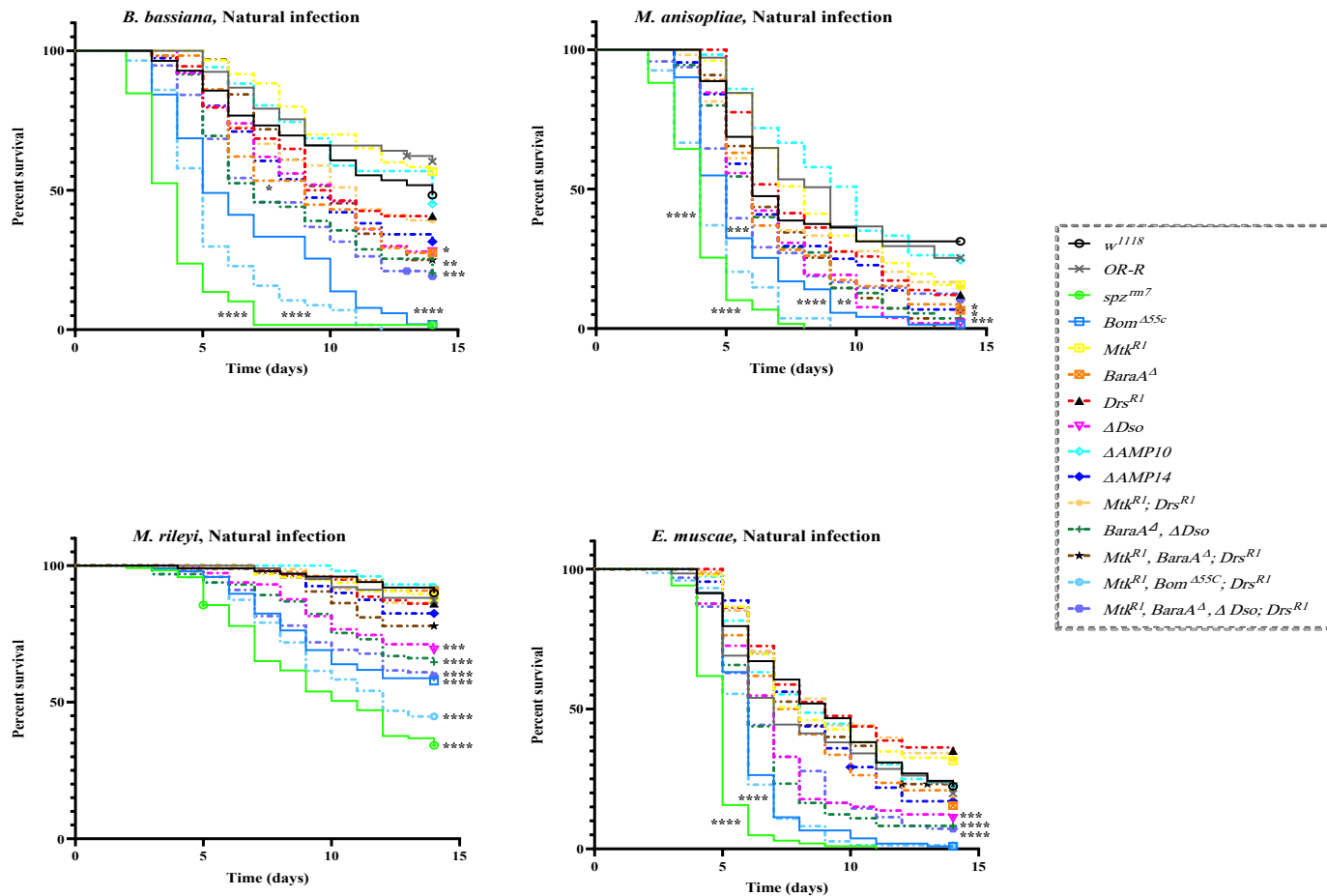

Supplement: S8 Fig — Full statistical details are available on S3 Table. Related to Fig 7. (PDF) [file ppat.1013995.s008.pdf]

Figure S10

A

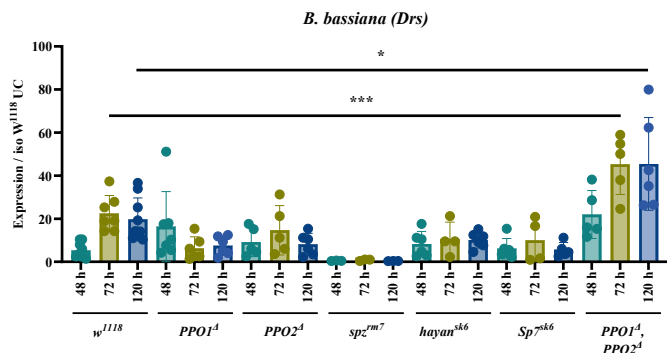

B

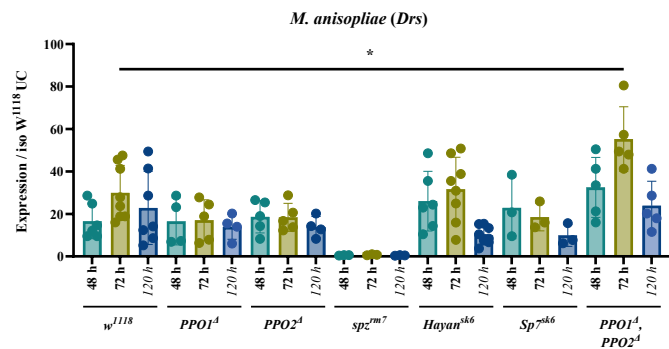

C

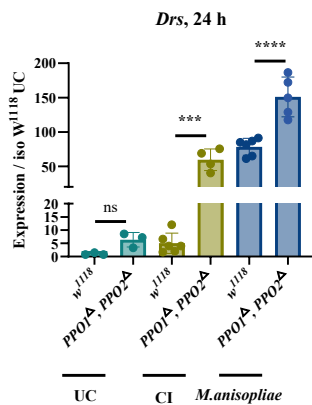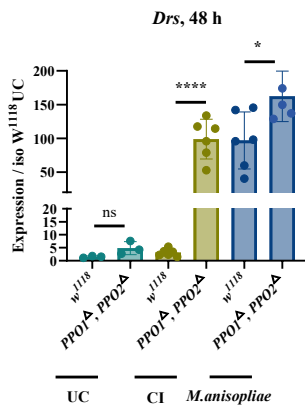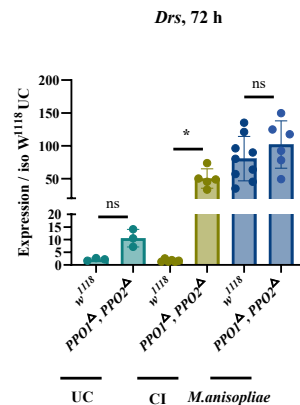

Supplement: S10 Fig — (A-B) Statistical analysis reveals higher Drosomycin expression in PPO1, PPO2 double mutant compared to wild-type at 72 h and 120 h after natural infection with B. bassiana and at 72 h after natural infection with M. anisopliae (*, P < 0.05; ***, P < 0.001). The spzrm7 flies as a positive control. (C) Compared with wild-type flies, PPO1, PPO2 double mutant flies exhibit an enhanced Toll pathway activity upon clean injury and septic infection with M. anisopliae (ns, P > 0.05; *, p < 0.05; ***, P < 0.001; ****, P < 0.0001). Expression was normalized with w1118 UC set as a value of 1. Data were analyzed using One-Way ANOVA followed by Tukey’s multiple comparison tests and values represent the mean ± s.d. of at least three independent experiments. Full statistical details are available on S3 Table. Related to Fig 7. (PDF) [file ppat.1013995.s010.pdf]
