# Supplementary material for: Drosophila host defense mechanisms against filamentous fungal pathogens with diverse lifestyles
Source: PLoS Pathog. 2026 Mar 23;22(3):e1013995. doi: 10.1371/journal.ppat.1013995 (PMC13035236; doi:10.1371/journal.ppat.1013995)
Supplement: S9 Fig — Full statistical details are available on S3 Table. Related to Fig 7. (PDF) [file ppat.1013995.s009.pdf]

Figure S9

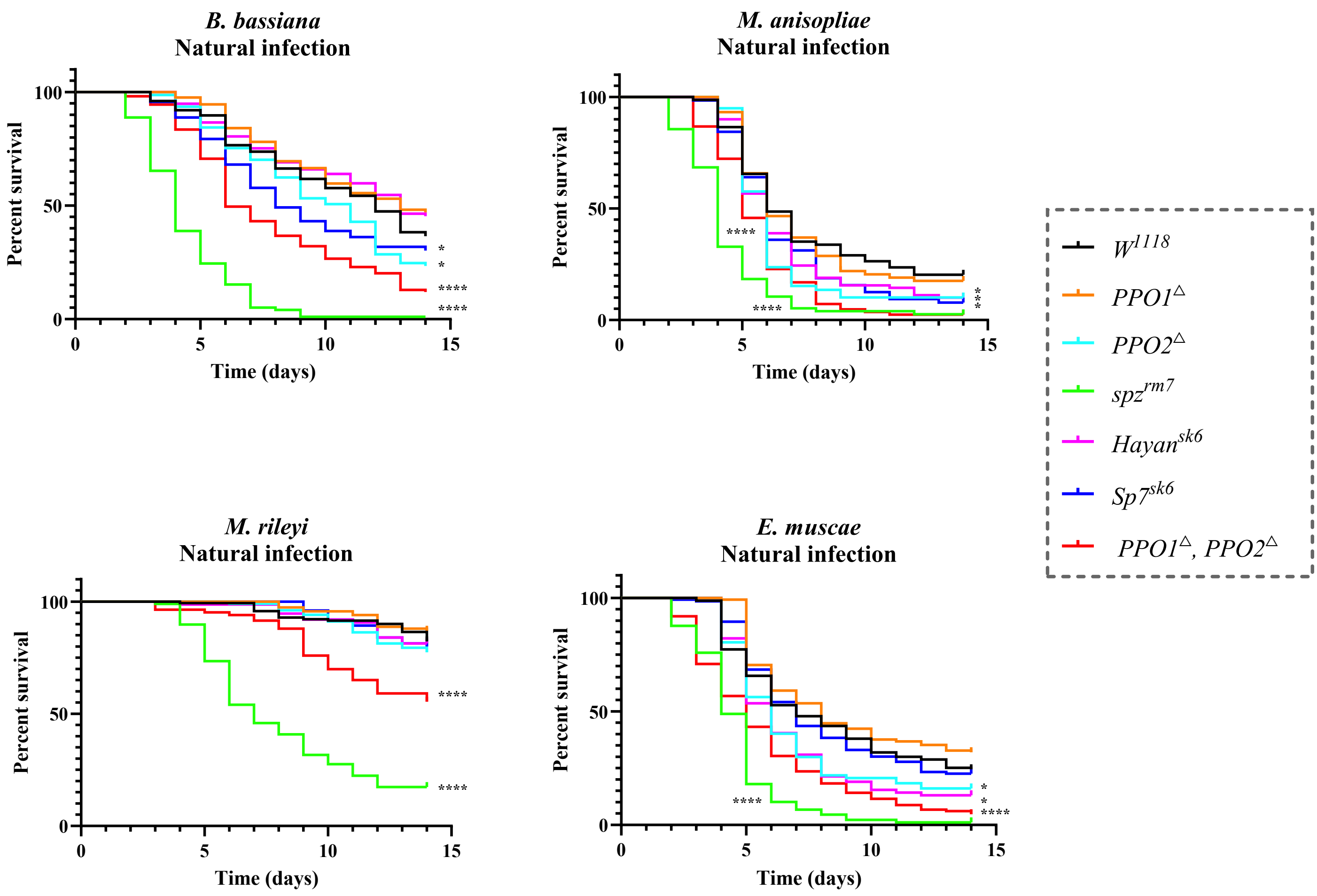
B. bassiana
Natural infection
Percent survival
Time (days)
M. anisopliae
Natural infection
M. rileyi
Natural infection
E. muscae
Natural infection
*
****
W1118
PPO1△
PPO2△
spzrm7
Hayansk6
Sp7sk6
PPO1△, PPO2△
