# Supplementary material for: Drosophila host defense mechanisms against filamentous fungal pathogens with diverse lifestyles
Source: PLoS Pathog. 2026 Mar 23;22(3):e1013995. doi: 10.1371/journal.ppat.1013995 (PMC13035236; doi:10.1371/journal.ppat.1013995)
Supplement: S1 Table — (DOCX) [file ppat.1013995.s011.docx]

**S1 Table** **List of wild-type and mutant flies used in this study.**

| ***Drosophila* lines** | **Source** | **Abbrevation** |
| --- | --- | --- |
| *iso; PPO1^△^; iso* | Binggeli et al., 2014 | *PPO1^△^* |
| *iso; PPO2^△^; iso* | Binggeli et al., 2014 | *PPO2^△^* |
| *iso; PPO1^∆^, PPO2^∆^; iso* | Binggeli et al., 2014 | *PPO1^∆^, PPO2^∆^* |
| *iso; iso; Rel^E20^* | Hedengren et al., 1999 | *Rel^E20^* |
| *iso; iso; spz^rm7^* | Lemaitre et al., 1996 | *spz^rm7^* |
| *+; Nim C1^1^; Eater^1^* | Melcarne et al., 2019 | *Nim C1^1^; Eater^1^* |
| *iso (Hayan-psh)^Def^; NimC1^1^; Eater1, Relish^E20^* | Ryckebusch et al., 2025 | *∆ITPM* |
| *Hayan^SK6^; iso; iso* | Duzic et al., 2019 | *Hayan^SK6^* |
| *psh**^SK1^; iso; iso* | Duzic et al., 2019 | *psh^SK1^* |
| *Hayan-psh^Def^; iso; iso* | Duzic et al., 2019 | *Hayan-psh^Def^* |
| *+; +; GNBP3△40/TM6B* | Gottar et al., 2006 | *GNBP3^hades^* |
| *iso; iso; GNBP3△* | Lu et al., 2024 | *GNBP3^△^* |
| *iso; GNBP like 3△; iso* | Lu et al., 2024 | *GL3^△^* |
| *iso; GNBP like 3△;GNBP3△* | Lu et al., 2024 | *G3^△^;GL3^△^* |
| *w[1118];Df(3L)ED4421,P{w[+mW.ScerFRT.hs3]* | BDSC#8066 | *ED4421^Def^* |
| *iso; iso; Sp7^SK6^* | Duzic et al., 2019 | *Sp7^SK6^* |
| *iso; iso; modSP^1^* | Buchon et al., 2009 | *modSP^1^* |
| *Psh^1^; iso; modSP^1^* | Buchon et al., 2009 | *psh^1^;; modSP^1^* |
| *iso; Mtk^R1^; iso* | Hanson et al., 2019 | *Mtk^R1^* |
| *iso; iso; Drs^R1^* | Hanson et al., 2019 | *Drs^R1^* |
| *iso; Bom[∆55C-289]; iso* | Clemmons et al., 2015 | *Bom^∆55C^* |
| *iso; BaraAΔ{dsRed}; iso* | Hanson et al., 2021 | *BaraA^Δ^* |
| *iso; ∆AMP10* | Caboni et al., 2022 | *∆AMP10* |
| *iso; ∆AMP14* | Caboni et al., 2022 | *∆AMP14* |
| *iso; ∆Daisho 1,2; iso* | Cohen et al., 2020 | *∆Dso* |
| *iso; Mtk^R1^; Drs^R1^* | Hanson et al., 2021 | *Mtk^R1^; Drs^R1^* |
| *iso; BaraA^SW1^, ∆Dso; iso* | Bruno Lemaitre | *BaraA^Δ^, ∆Dso* |
| *iso; Mtk^R1^, BaraA^SW1^; DrsR1* | Bruno Lemaitre | *Mtk^R1^,BaraA^Δ^; Drs^R1^* |
| *iso; Mtk^R1^, BaraA^SW1^, ∆Dso; DrsR1* | Bruno Lemaitre | *Mtk^R1^,BaraA^Δ^,∆Dso; Drs^R1^* |
| *iso; Mtk^R1^, Bom^∆55C^; Drs^R1^* | Bruno Lemaitre | *Mtk^R1^, Bom^∆55C^; Drs^R1^* |
| *w;; Hml^P2A^-GAL4* | Stephenson et al., 2022 | *Hml^P2A^-GAL4* |
| *w;; UAS-CaMPARI2* | Moeyaert et al., 2018 | *UAS-CaMPARI2* |
| *w; HmlΔ GAL4,UAS GFP;* | Defaye et al., 2009 | *Hml GAL4,UAS GFP* |
| *w;UAS-Bax;* | Gaumer et al., 2000 | *UAS Bax* |
