# Supplementary material for: Drosophila host defense mechanisms against filamentous fungal pathogens with diverse lifestyles
Source: PLoS Pathog. 2026 Mar 23;22(3):e1013995. doi: 10.1371/journal.ppat.1013995 (PMC13035236; doi:10.1371/journal.ppat.1013995)
Supplement: S2 Table — (DOCX) [file ppat.1013995.s012.docx]

**S2 Table Primers used in this study.**

| **Genes** | **Primers** | **Primer sequencs (5'-3')** | **Application** | **Source** |
| --- | --- | --- | --- | --- |
| Fly GNBP3 | G3-F | TGGCGTTCAGGGGTATGAAG | Identify GNBP3 mutant | Lemaitre lab |
|  | G3-R | CGTCTTCGCGATAACCCAGT |  |  |
| Fly *Drs* | Drs_F | CGTGAGAACCTTTTCCAATATGAT | RT-PCR/qPCR | Lemaitre lab |
|  | Drs_R | TCCCAGGACCACCAGCAT |  |  |
| Fly *BomBc3* | BomBc3_F | CTGATCGGCGCTCATCCCAG | RT-PCR/qPCR | Lemaitre lab |
|  | BomBc3_R | GGGATGAGGAGAAGCTGCGG |  |  |
| Fly *Rp49* | Rp49_F | GCCGCTTCAAGGGACAGTATCTG | RT-PCR/qPCR | Lemaitre lab |
|  | Rp49_R | AAACGCGGTTCTGCATGAG |  |  |
| *B. bassiana 18S rRNA* | Bbas_18S_F | CGGGTAACGGAGGGTTAGG | Detection of fungal load | Hasan et al., 2021 |
|  | Bbas_18S_R | AGTACACGCGGTGAGGCGG |  |  |
| Fly *actin* | Act-F | GCCAACCGTGAGAAGATGA | Detection of fungal load | Shang et al., 2023 |
|  | Act-R | GGTGGTGAAAGAGTAACCGC |  |  |
| *M. anisopliae Rpl32* | Rpl-F | ATCGGTTTCGGGTCCAAC |  |  |
|  | Rpl-R | ATTCGCTTTCGGGAGGAG |  |  |
| *E. muscae* 18SrRNA | Emus_18S_F | CGGGTGACGGGGAATAAGG | Detection of fungal load | Lemaitre lab |
|  | Emus_18S_R | GCCAAGAACTGAAACTAACCC |  |  |
