# Supplementary material for: Drosophila host defense mechanisms against filamentous fungal pathogens with diverse lifestyles
Source: PLoS Pathog. 2026 Mar 23;22(3):e1013995. doi: 10.1371/journal.ppat.1013995 (PMC13035236; doi:10.1371/journal.ppat.1013995)
Supplement: Attachment — Submitted filename: 161049_1_rebuttal_3640303_t906j9.pdf [file ppat.1013995.s014.pdf]

Full Revision

Review COMMONS

Manuscript number: RC-2025-03183
Corresponding author(s): Guiqing Liu and Bruno Lemaitre

1. General Statements [optional]

Dear editor,

Thank you very much for handling our manuscript. We greatly appreciate the reviewers' comments and are pleased that their evaluations of our work were very positive. Below, we provide a point-by-point response detailing the revisions we have made, including textual changes and additional experiments, to address all of the points raised by the reviewers.

With best regards,

Guiqing Liu & Bruno Lemaitre

2. Point-by-point description of the revisions

This section is mandatory. Please insert a point-by-point reply describing the revisions that were already carried out and included in the transferred manuscript.

Reviewer #1 (Evidence, reproducibility and clarity (Required)):

The key conclusions are solid. All the claims are supported by quality data. The content is rich, and no additional experiment is needed. The data and methods are properly presented for reproduction. The experiments are adequately replicated. One comment on statistical analysis is listed below.

Summary:

This manuscript investigates how Drosophila immune pathways contribute to defense against a range of filamentous fungi with distinct ecological

strategies. The work provides novel insights into Toll pathway activation through pattern recognition receptors and danger signals, relative roles of melanization, phagocytosis, and effects of antimicrobial peptides, and particularly the immune evasion strategy of E. muscae via protoplast formation. These findings are of broad relevance to insect immunology, host-pathogen interactions, and evolutionary biology.

The study is well designed, the experiments are carefully executed, and the manuscript is clearly written. It is novel to demonstrate that E. muscae evades immune recognition via protoplast formation. However, some aspects of clarity and discussion of limitations could be improved before publication.

We thank the reviewer of the positive assessment of our manuscript.

Major comments:

1) The Abstract is informative but a bit too long. Consider condensing some sentences and highlighting the novel contributions (e.g., role of protoplasts in immune evasion.).

Good points. We have reduced the abstract. The sentence is *'Our study also reveals that the fly-specific obligate fungus Entomophthora muscae employs a vegetative development strategy, protoplasts, to hide from the host immune response.'*

We believe that the role of protoplasts is already mentioned in the abstract.

2) The Results may use more mechanistic links. For instance, the section on E. muscae immune evasion could more explicitly connect the morphological findings (protoplasts, lack of cell wall) with specific immune recognition failures.

Our article is a comparison of Drosophila host defense against fungi with various life styles. This obviously complexify the presentation of the results. We have made the maximum of effort to explain our data with clarity. We believe that having two successive sections entitled '**Natural infection with E. muscae barely induces the Toll pathway**' followed by ' **Entomophthora muscae hides from the host immune response using a vegetative development strategy'** expose well the idea that E. muscae has a specific hiding strategy. We did not change this part.

3) Please clarify statistical analyses used for survival data (e.g., log-rank tests, multiple testing corrections).

We have clarified the statistical analysis in the method part. The sentence is 'Statistical significance of survival data was calculated with a log-rank test (Mantel-Cox test) comparing each genotype to w^{1118} flies'.

Minor comments:

Abstract: 1) "The infection outcome depends on the complex interplay between insect immune defenses and fungal adaptive strategies." could be simplified to: "Infection outcomes depend on the interplay between insect immunity and fungal adaptation." 2) Replace "our study uncovers" with "we show" for more concise phrasing. Reduce phrases like "our study reveals" or 'we conclude" in other parts of the manuscript.

Results: p. 5: phrase "survival upon natural infection… reveals the major contribution" → reword to avoid passive tone. p. 10: clarify "vesicles push the membrane outwards" with more precise terminology (e.g., budding,

extrusion).

Discussion: p. 20: streamline sentence beginning "These observations provide a mechanistic basis..." (currently too dense).

We have taken in consideration all these comments. Note that we removed in the revised version the sentence "The infection outcome depends on the complex interplay between insect immune defenses and fungal adaptive strategies." To shorten the abstract, we have removed the sentence 'These observations provide a mechanistic basis for future exploration.'

Referee cross-commenting

I agree with the comments of the other two reviewers.

Reviewer #1 (Significance (Required)):

This manuscript investigates how Drosophila immune pathways contribute to defense against a range of filamentous fungi with distinct ecological strategies (generalists, specialists, opportunists). By leveraging a comprehensive panel of genetically defined fly lines and standardized infections, the authors provide a demonstration that the Toll pathway is the predominant systemic antifungal defense, extending classical findings into a comparative framework across fungal lifestyles. The work provides novel insights into Toll pathway activation through GNBP3 and fungal proteases sensed by Psh, while also dissecting the relative contributions of melanization, phagocytosis, and antimicrobial peptides to host protection.

Of particular note is the compelling demonstration that the fly specialist E. muscae can evade immune recognition through protoplast-like vegetative forms, minimizing cell-wall exposure and thereby escaping Toll activation.

My expertise and limitations:

Insect biochemistry and molecular biology, with particular focus on innate immunity, serine protease cascades, melanization, and host-pathogen interactions. I also have experience with genetic, biochemical, and functional approaches to dissecting immune signaling pathways in model insects. However, I do not have sufficient expertise to critically evaluate advanced statistical analyses.

Reviewer #2 (Evidence, reproducibility and clarity (Required)):

In this work the authors describe the contribution of distinct immune responses in Drosophila melanogaster to systemic and natural infections with 5 fungal species with different lifestyles some being generalists infecting a broad range of insects while others being more specialists or opportunistic. The authors used several well characterized Drosophila mutants of the Toll, Imd, phagocytosis and melanization responses to address this question. They show that Toll pathway is the key player in anti-fungal resistance in both natural and septic infections, whereas melanization plays a minor role mainly during natural infections possibly to limit fungal invasion through the cuticle. The authors show elegantly using different combinations of mutants for antimicrobial peptides genes with

antifungal activities that Bomanins and Daisho (1 and 2) are the main Toll effectors mediating resistance to fungi but the authors did not find specific fungus-by-gene interaction, but rather antifungal peptides seem to act in a more general fashion against the fungi tested with significant redundancies between certain classes. Interestingly the authors show that while generalists like Beauveria and Metarhizium strongly activate the Toll pathway, the specialist E. muscae weakly activates the pathway and the opportunistic A. fumigatus[1] does not activate the pathway, indicating that certain fungal species are able to evade sensing by immune pathways. In the context of the Toll activation, the sensor protease Psh and not GNBP3 seem to be the main trigger of the pathway.

Minor comments

This is an interesting work that compares the contributions of different arms of the fly immune response to 5 fungal species with diverse lifestyles. The use of different lines with different combinations of mutant genes is a strength to highlight the relative contribution of each immune response. Some of the data obtained is intriguing and warrants more future investigations such as the distinct phenotypes of ModSp and GNBP3 mutants in E. muscae infections. The methodology is robust and the conclusions are supported with good experimental evidence. I do not see any major concerns with the work. I just have some minor comments listed below

[1] Check that this is includes in the abstract

We thank the reviewer for the positive comments on our manuscript.

1- Statistical significance should be indicated on Figures 1 and 2, although it appears in the legend.

We have added statistical significance on Figures 1 and 2.

2- It is not very accurate to use the term resistance of the different mutants to infections with the diverse fungal species in Figures 1 and 2 especially that the authors have reported only survival data in these figures and have not measured fungal proliferation in infected flies (although they did that in later figures). It is more accurate to mention that the mutants flies have different levels of tolerance rather than resistance to fungal infections.

We agree that we cannot use the term 'resistance' in Figures 1 and 2, since this term has now a more restricted meaning in the community. We have replaced the term 'resistance' by 'host defense' or 'surviving' through the text to avoid the confusion, except when the bacterial load was monitored.

3- The authors show that Toll is over-activated in PPO1/PPO2 double mutant possibly through a negative feedback mechanism. However, there could be another explanation for this observation: For instance, the increased fungal proliferation in the PPO double mutant results in increased protease secretion by fungi enhancing Psh activation! Also, how can fungi manage to proliferate in this double mutant if Toll is overactivated? Could it be that Toll overactivation is triggering a fitness cost?

The reviewer raises a good point. It is difficult to reconcile the susceptibility of PPO1/2 mutants to fungi taking in consideration the higher Toll activation. The higher activation of Toll could be deleterious and We clearly observed higher Toll pathway activation in PPO1/2 flies upon clean injury (Fig. S9C) or injection of dead spores (data not shown). Thus, this higher expression cannot be only explained as a consequence of higher fungal growth.

4- In Lines 654-655, it is not accurate to say that E. muscae protoplasts are not detected by the immune response since E. muscae natural infections triggers Drs expression at 24 hpi and there is possibly some melanization taking place since PPO1 and PPO2 are required for defense against this fungus. A more accurate explanation is that this fungus is possibly more resistant to the effectors of the host immune response than the other fungi. I think a major point that the authors might have missed to consider in the discussion of their data is that the different fungi used herein may exhibit different levels of resilience to the effector reactions of the host such as AMPs and melanin deposition

The observation that injection of E. muscae protoplasts do not trigger an immune response above the level of clean injury is a strong argument that support our view that E. muscae protoplasts are not immunogenic. The reviewer is correct by underlying the small but significant induction of Drs at 24h post natural infection. We hypothesize that this could be due to mechanical injury associated with the entry of E. muscae. We have added a sentence to underline the possibility raised by the reviewer: 'Although we

cannot rule out that the high pathogenicity of E. muscae may be partly due to the fungus's increased resilience, we favor the interpretation that it is instead mainly driven by its capacity to evade immune detection.'

Reviewer #2 (Significance (Required)):

Although the importance of Toll pathway and melanization in antifungal immunity is not new per se, this work adds to this knowledge by showing that Toll has the upper hand in anti-fungal immunity and that the strength of Toll pathway activation and its effector capacity may vary depending on the type of invading fungus. The work also highlights that certain fungi may employ a delayed switch to hyphal growth to reduce the presence of cell wall sugars as a mechanism to evade immune recognition. Overall, this work significantly adds to the knowledge of Drosophila immunity and raises some interesting questions related to the evolution of host-pathogen interactions and to the complex functions of serine protease cascades regulating Toll and melanization. This work will be of interest to a broad audience in the field of host-pathogen interactions

Reviewer #3 (Evidence, reproducibility and clarity (Required)):

This is a clearly written manuscript on the immune effector mechanisms regulating Drosophila melanogaster host defense against a broad range of fungal pathogens, including entomopathogenic and saprophytic filamentous fungi. The authors systematically dissect the contribution of major arms of

Drosophila immunity, including cellular and humoral responses and melanization and potential mechanisms of cross talk using genetic tools and reporter lines. They also go into detail to characterize the contribution of upstream activators of these responses by fungal PAMPs and the role of antimicrobial effectors (AMPs) in fly susceptibility.
They conclude for no important role of phagocytosis in host defense. Instead, they find important contributions of Toll pathway mainly through the detection of fungal proteases by Persephone rather than b-glucan detection by GNBP3. They also demonstrate that Toll activation is proportional to the virulence of the fungal pathogen, showing little activation of this response by Aspergillus fumigatus. Finally, they identify melanization as another line of host defense that restricts pathogen dissemination and protects fly from invasive fungal disease. A very interesting part of this study is the identification of a virulence strategy of the obligate fungus Entomophthora muscae, which employs a vegetative development strategy, by making protoplast that avoid immune recognition by masking immunostimulatory cell wall molecules to avoid immune recognition by Toll pathway until the very last stage of invasive growth. Overall, this is a very interesting study on host-pathogen interplay in Drosophila, shedding light onto novel pathogenetic mechanism employed by entomopathogenic fungi to adapt to their hosts.
We thank the reviewer for his positive assessment.

Major comments for the authors:

1. The use of reporter fungal strains to capture the dynamic interplay of

the pathogen and the different arms of the immune system precludes firm conclusions on the contribution of various immune response to infection. This should be emphasized in the discussion

Unfortunately, we did not fully understand this point. Note that we monitored both survival and when possible fungal load (*B. Beauveria, E. muscae* and *M. anisopliae* for Toll; and *B. Beauveria*, and *M. anisopliae* for melanization) allowing to state that Toll and Melanization are contributing to host defense by limiting fungal growth.

2. The route of infection and the method employed to inject fungal spores has an impact on the effector pathways being activated. For example, pricking introduces spores less efficiently in the hemolymph compared to microinjection. The inoculum size in case of microinjection also has profound impact in understanding the role of cellular and humoral immunity during the infection course. For example, the lack of Toll activation in the natural infection with A. fumigatus does not mean that this pathway is not important in host defense against this pathogen.

We fully agree and expected to clarify this different outcome between septic injury and natural infection. In the case of A. fumigatus, we confirm that Toll is important upon systemic infection but not natural infection because this fungus has a limited ability to penetrate insect by the natural route. We have clarified this in the text by adding the sentence: *'The low Toll pathway activation by A. fumigatus is likely* due *the weak ability of this fungus to penetrate insect by the natural route.'*.

3. The use of total KO strains does not preclude the cross talk of cellular and humoral immunity and consequently potential defects in cellular immunity upon deletion of a master regulator of the Toll pathway or even its downstream effectors

The observation that Toll deficient mutants are almost as susceptibility as mutant flies lacking all the four immune modules (△ITPM) to the five fungal pathogens point to a major role of this pathway. In a previous study (Ryckebusch et al Elife 2025), we have shown that the four immune pathways largely work independently as phagocytosis was still observed in Toll deficient mutant.

4. Did the authors validate that NimC11; Eater1 flies are not able to phagocytose fungal spores?

In the first version of this manuscript, we did not validate that *NimC1;eater* flies are phagocytic deficient also for Fungal spores although our manuscript assumed it. To address the comment of the reviewer, we have extended our study to better characterize the role of the cellular immune response to fungal infection (See new Figure S1).

Our new results show that *NimC1;eater* deficient flies have defect in binding to *M. anisopliae* GFP spores (New Supplement Figure S1E,F). We did not see clear evidence of internalization. Thus, we conclude that the use of *NimC1;eater* flies is adequate to study the role of the cellular response. We have monitored the survival of *hemoless* flies that lack nearly all plasmatocytes due to the over-expression of the proapoptotic gene Bax, to

natural infection and septic injury with B. bassiana and M. anisopliae. This new piece of data (described in New Supplementary Figure S1A-D) show that hemoless flies display a wild-type survival to B. Bassiana and a mild susceptibility to M. anisopliae consistent with our previous statement that the cellular response is less important than the humoral response. In the revised version, we have added this new piece of data and nuanced our statement on the role of the cellular response to fungal infection.

5. Is it possible that entomopathogenic fungi bypass phagocytosis as a virulence strategy by inducing large size germinating cells, which are not phagocytosed?

Indeed, there are several studies have showed that entomopathogenic fungi have evolved sophisticated strategies to evade or survive phagocytosis.

1) Once fungal spores (conidia) germinate, penetrate host tegument and reach the hemocoel, fungi exist within the hemocoel in the forms of blastospores with thinner cell walls than conidia (M. anisopliae, M. rileyi, B. bassiana), and cell wall-free protoplasts (E. muscae). Wang and St Leger (2006) had demonstrated that host hemocytes can recognize and ingest conidia of M. anisopliae, but this capacity is lost on production of blastospore, because of its ability to avoid detection depending on the cell surface hydrophobic protein gene Mcl1 that is expressed within 20 min of the fungal pathogen contacting hemolymph.
2) Other studies have shown that blastospores of B. bassiana and M. anisopliae can be phagocytosed at the early stages of infection but

manage to emerge from host cells and continue to propagate. Growing hyphal bodies can deform the plasmatocyte cell membrane (Gillespie et al., 2000; Hung and Boucias, 1992; Vilcinskas et al., 1997). Studies have also shown that during the infection process of entomopathogenic fungi in insects, the hemocyte count gradually decreases. For instance, during the infection of *Thitarodes xiaojinensis* by *Ophiocordyceps sinensis*, blastospores are the initial cell type present in the host hemocoel and remained for 5 months or more before transformation into hypha, which finally led to host death; and the increase in blastospores quantity coincidence with a decline in hemocyte count (Liu et al., 2019; Li et al., 2020).

In a new set of experiments, we tested the ability of plasmatocytes to phagocytose *M. anisopliae*–GFP spores. We observed that plasmatocytes bind to the spores, but we did not obtain clear evidence of internalization (New Figure S1E,F). However, this assay was not sufficient to conclusively determine whether plasmatocytes internalize *M. anisopliae* spores, as GFP fluorescence may be quenched in acidic intracellular compartments. Because entomopathogenic fungi can affect hemocyte abundance, we also monitored the expression level of *Hml*, a hemocyte-specific marker, in flies following natural infection with *B. bassiana*, *M. anisopliae*, *M. rileyi*, and *E. muscae* at 2, 3, and 5 days post-infection (see figure below). We did not observe a reduction in hemocyte levels for any of these fungi except *M. anisopliae*. This suggests that *M. anisopliae* may reduce hemocyte numbers as a strategy to circumvent the cellular immune response. These results, although promising, were not included in the revised version of the manuscript, as a thorough analysis of the cellular immune response would require a dedicated study on its own.

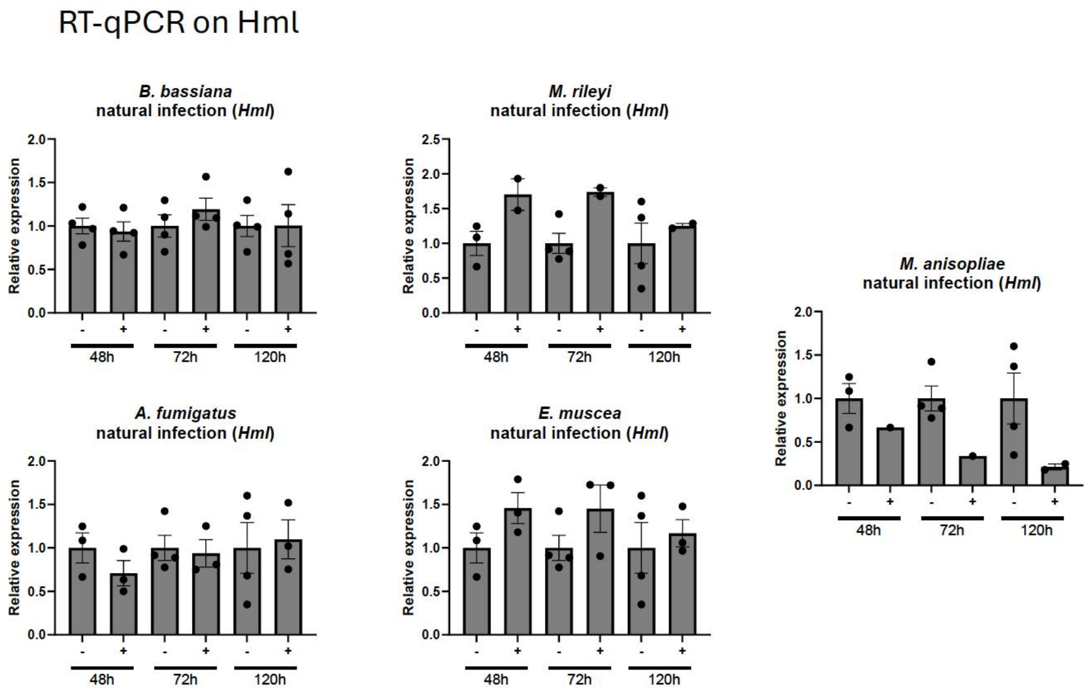

Figure: Expression of Hml by RT-qPCR upon natural infection with entomopathogenic fungi (figure not included in the revised manuscript)

6. Is it possible that fungal toxins kill phagocytes during germination?

There are indeed evidences that fungal toxins destruxins (DTXs) induce ultrastructural alterations of circulating plasmatocytes and sessile haemocytes of Galleria mellonella larvae. DTXs contribute to the fungal infection process by a true immune-inhibitory effect. This is evidenced by two key findings: first, the germination rate of injected Aspergillus niger spores was slightly but significantly enhanced; second, during incubation, the fungus demonstrated a greater ability to escape from the haemocyte-formed granuloma envelope (Vilcinskas et al., 1997; Vey et al., 2002). But in Drosophila, Destruxin does not appear to affect Drosophila cellular

immune responses in vivo. Phagocytosis of E. coli bacterial particles in Destruxin-injected flies appeared to be the same as that seen in PBS-injected flies. The proliferation of bacteria in the Destruxin-injected flies was due to the lower expression of antimicrobial peptide genes suggesting that Destruxin A specifically suppressed the humoral immune response in Drosophila (Pal et al., 2007), which is consistent with major role of antimicrobial peptides in survival to fungi.
This point is now discussed in the discussion with a new section on the cellular response to fungal infection.

Reviewer #3 (Significance (Required)):

This is an important work that provide new information on virulence mechanisms of entomopathogenic fungi and the host immune responses that mediate host protection. The authors should address my comments in the discussion and provide some additional evidence by using reporter fungal strains for hemocytes on whether these fungal pathogens completely bypass phagocytosis to invade the host. Therefore, rather than claiming that phagocytosis is not important it should be clarified whether phagocytes are directly involved in host defense or whether the fungus changes its cell wall surface to avoid this line of host defense. My expertise is on phagocyte biology and host-fungal interaction on human fungal pathogens.

We have added more information showing that plasmatocytes of *NimC1;eater* larvae fail to bind to spores of *M. anisopliae* suggesting that

this line provides an appropriate tool to assess phagocytosis. We have also analyzed the survival of flies depleted for plasmatocytes via the over-expression of bax, which revealed a mild role for plasmatocyte in defense against *M. anisopliae* but not *B. bassiana*. By performing additional experiments, we realized that analyzing the role of cellular immunity in host defense against these five fungi would require much more work and is beyond the scope of this study. We have however added in the revised version a para in the discussion on the the cellular response.
